# The relationship between work addiction and addictions to social media, shopping, food, caffeine, and nicotine

**DOI:** 10.1080/00049530.2025.2486774

**Published:** 2025-04-02

**Authors:** Emrah Özsoy, Mark D. Griffiths, Gülay Tınmaz Karaçay, Ömer Alperen Onay, Canan Yılmaz, Özlem Balaban

**Affiliations:** a Sakarya Business School, Sakarya University, Sakarya, Türkiye; b International Gaming Research Unit, Psychology Department, Nottingham Trent University, Nottingham, UK; c Faculty of Economics, İstanbul University, İstanbul, Türkiye; d Independent Researcher, Ankara, Turkey

**Keywords:** Work addiction, behavioural addictions, social media addiction, shopping addiction, food addiction, caffeine addiction, nicotine addiction, addiction comorbidity

## Abstract

**Purpose:**

Work addiction is a relatively underexplored behaviour compared to other forms of addiction. Existing research predominantly focuses on the antecedents and consequences of work addiction. However, studies examining its relationship with other types of addiction are notably limited. Therefore the present study investigated the relationships between work addiction and five other types of addiction (i.e. social media addiction, shopping addiction, food addiction, caffeine addiction, and nicotine addiction).

**Method:**

The research was conducted with 693 employees working in both public and private sectors. Data were collected through an online survey comprising validated scales for assessing the specific types of addiction and demographic questions. Descriptive statistics, internal consistency tests and Pearson correlation analysis were employed for data analysis.

**Results:**

Correlation analysis showed significant (albeit weak) positive relationships between work addiction and food addiction (*r* = .14), shopping addiction (*r* = .12), and caffeine addiction (*r* = .16). However, no significant relationships were found between work addiction and social media addiction or nicotine addiction. Comorbidity among individuals classified as high-risk for more than one addiction was only observed between two substance-based addictions (caffeine and nicotine), and between one substance-based addiction (caffeine) and one behavioural addiction (social media).

**Conclusions:**

These findings suggest that although multiple behavioural addictions may be associated, the observed comorbidity patterns primarily occur between two substance-based addictions or between a substance-based and a behavioural addiction, rather than between two behavioural addictions.

## KEY POINTS

**What is already known about this topic**:

(1) Work addiction may be associated with other types of addiction.
(2) The relationship between work addiction and social media addiction has been studied, but its association with other addictions remains under explored.
(3) Addictions are known to share common psychological and behavioural foundations.

**What this study adds**:

(1) A weak but significant positive relationship was found between work addiction and (i) food addiction, and (ii) caffeine addiction.
(2) High-risk group overlap was primarily observed between two substance-based addictions or between a substance-based and a behavioural addiction.
(3) The likelihood of two behavioral addictions (e.g. work addiction and social media addiction) co-occurring in the same high-risk individual was found to be low.

## Introduction

Work addiction was first defined by Oates (1968) and has been a subject of academic research for decades. Work addiction is characterized by excessive concern about work, an uncontrollable drive to work, and devoting too much time and energy to work, negatively impacting social relationships, leisure activities, mental health, and physical well-being, along with a persistent prioritization of work and mental preoccupation with it (Andreassen, 2014a; Andreassen et al., 2012).

Although the terms “work addiction” and “workaholism” are often used interchangeably, there are distinctions between the two concepts (Griffiths, 2024a; Griffiths et al., 2018). While encompassing long working hours and excessive and compulsive work behaviours, workaholism can also include enjoyment of work and positive experiences during the process (Schaufeli et al., 2008; Spence & Robbins, 1992). However, like other forms of addiction, work addiction can lead to clinically adverse outcomes (Griffiths, 2011) and has detrimental effects on individuals, organizations, and social environments (Griffiths, 2024b). For this reason, workaholism is considered a broader concept than work addiction. Every individual with work addiction could be classified as a workaholic; however, not all workaholics necessarily exhibit the clinical characteristics of work addiction (Griffiths, 2024a; Griffiths et al., 2018; Kızıloğlu et al., 2024). The primary reason for emphasizing this distinction in the present study is that work addiction is examined alongside other types of addiction and shares common psychological and behavioural mechanisms with them. Moreover (as aforementioned), work addiction is distinct from workaholism, because unlike work addiction, workaholism does not always have negative consequences and can lead to beneficial outcomes (Griffiths et al., 2018).

Research examining work addiction has predominantly focused on its antecedents (Kızıloğlu et al., 2024; Kun et al., 2021) and consequences (Fekih-Romdhane et al., 2022; Özsoy & Balaban, 2024). However, the extent to which work addiction overlaps with other types of addiction remains insufficiently explored. To date, studies have primarily focused on the relationship between work addiction and various technology-based addictions such as internet addiction (Quinones et al., 2016) and social media addiction (Horváth et al., 2024). There is a lack of research examining the overlaps between work addiction and other forms of addiction, such as shopping addiction, food addiction, nicotine addiction, and caffeine addiction. On the other hand, relationships between other types of addiction, such as social media addiction and gaming addiction (Yayman & Bilgin, 2020) or food addiction and substance use (Bonder & Davis, 2022), have been investigated. Nevertheless, studies that include work addiction in the exploration of overlaps among different addiction types remain few.

Due to the shared psychological, behavioural, and neurobiological foundations of addictive behaviours, overlaps can be observed among different types of addiction (Kótyuk et al., 2020). On a psychological level, factors such as loss of control (Robinson & Berridge, 2003), personality traits such as negative affectivity and low self-esteem (Kun et al., 2021), and psychopathological elements such as depressive symptoms and compulsivity (Andreassen, Griffiths, et al., 2016; Fekih-Romdhane et al., 2022; Hussain & Griffiths, 2018), are associated with addictive behaviours. On a behavioural level, conditioned learning processes (Everitt & Robbins, 2005) play a significant role in reinforcing addictive behaviours. At the neurobiological level, reward sensitivity within the dopamine system (Koob & Volkow, 2016) and hypodopaminergic characteristics (Blum et al., 2000) are highlighted as common mechanisms underlying many different types of addiction.

The present study explored the overlapping aspects of work addiction with addictions to social media, shopping, food, nicotine, and caffeine. A large review of 11 different types of addiction suggested that higher levels of work addiction are likely to be associated with an increased tendency to engage in these addictive behaviours (Sussman et al., 2011), and it has also been suggested that one type of addiction may potentially trigger others (DiNicola et al., 2015). By investigating the relationships between work addiction and other forms of addiction, the present study sought to examine a more nuanced perspective on the shared mechanisms underlying addictive behaviours.

### Hypotheses development

#### Work addiction and social media addiction

Social media has been defined as *“Internet-based, disentrained, and persistent channels of masspersonal communication facilitating perceptions of interactions among users, deriving value primarily from user-generated content”* (Carr & Hayes, 2015, p. 49). Social media addiction is an uncontrollable urge to engage in online social media activities along with an excessive investment of time and effort in social media, neglecting other significant areas of life (Andreassen & Pallesen, 2014). The integration of digital platforms into work life may increase the tendency of individuals with high levels of work addiction to monitor work-related updates in digital environments compulsively. This behaviour may manifest particularly in following work-related updates, and maintaining professional correspondence (Horváth et al., 2024). Because individuals with high levels of work addiction tend to remain engaged with work even outside of working hours (Clark et al., 2020), the constant accessibility provided by digital environments may further hinder their ability to detach from work (Quinones et al., 2016). In addition, individuals with high levels of work addiction may exhibit a greater tendency to use their smartphones frequently (typically the main device on which individuals access social media sites) to avoid missing important opportunities, information, or events (Cheung et al., 2022; Spagnoli et al., 2019). Due to the widespread use of smartphones for accessing social media platforms, these individuals are likely to increase their social media use to track work-related content. Moreover, unsatisfied psychological needs (e.g., love, respect, validation, and recognition) are among the individual antecedents of work addiction (Andreassen et al., 2010; Özsoy, 2019a). These psychological deficiencies may increase individuals’ desire to showcase their professional achievements on social media (Krasnova et al., 2015), ultimately leading them to enhance their reputation or strengthen their self-image (Falco et al., 2017). Based on these considerations, it was hypothesized that work addiction would be positively associated with social media addiction (H_1_).

#### Work addiction and and shopping addiction

Shopping addiction is the uncontrollable motivation to shop, where individuals spend excessive time and effort on shopping, thereby affecting other significant life domains (Andreassen, 2014b). Individuals with high levels of anxiety and depression are more prone to shopping addiction, often engaging in shopping as a means to escape emotional distress (Rachubińska et al., 2022). Individuals with a high level of work addiction may to turn to shopping to alleviate the tension (Sussman & Sussman, 2011) caused by high levels of work stress (Özsoy & Balaban, 2024). The accessibility provided by digital platforms facilitates shopping for work-addicted individuals, allowing them to experience short-term pleasure and momentary relief (Weinstein & Lejoyeux, 2010). Additionally, shopping can help individuals shift their focus when under stress and make them feel happy by purchasing a desired product (Atalay & Meloy, 2011; Zheng & Ma, 2021). It can also help individuals experience a sense of control and autonomy during the decision-making process and derive satisfaction from purchasing (Rick et al., 2014). These psychological benefits can make shopping a coping mechanism for managing work-related stress or other negative emotions. At its core, shopping addiction is less about shopping itself and more about the pleasure and satisfaction derived from the act (Arnold & Reynolds, 2003). Similarly, work addiction is characterized by the satisfaction and sense of accomplishment obtained from work, often leading to a loss of control (Andreassen et al., 2014). In this context, both types of addiction are related to individuals’ pursuit of pleasure to alleviate stress and emotional discomfort. The digitalization of shopping processes may further strengthen the possible connection between these two types of addiction. Therefore, it was hypothesized that work addiction would be positively associated with shopping addiction (H_2_).

#### Work addiction and food addiction

Food addiction is an abnormal eating behaviour characterized by symptoms such as loss of control over eating, persistent cravings, and continued consumption despite adverse consequences (Fekih-Romdhane et al., 2022; Hauck et al., 2020; Meule & Gearhardt, 2014). Work addiction and food addiction may share common psychological comorbidities (e.g., depression, stress, anxiety), behavioural symptoms (e.g., compulsive engagement in the addictive behaviour, cravings, and withdrawal symptoms), and neurobiological mechanisms (e.g., dysregulation of the brain’s reward and motivation systems) (Fekih-Romdhane et al., 2022; Hauck et al., 2020). In this context, individuals addicted to work may engage in eating behaviours to achieve emotional relief under intense work-related stress. Importantly, individuals addicted to work may struggle to find sufficient time for meal preparation due to their demanding schedules, which can lead to a preference for convenience foods or snacks, thereby reinforcing this behaviour. It is argued that individuals experiencing one of these addictions may be more susceptible to developing the other, particularly in the context of emotional dysregulation and stressful life conditions (Fekih-Romdhane et al., 2022). Therefore, it was hypothesized that work addiction would be positively associated with food addiction (H_3_).

#### Work addiction and caffeine addiction

Caffeine, a stimulant commonly found in natural products such as coffee beans, cocoa beans, and tea leaves, affects the central nervous system and is widely consumed (de Mejia & Ramirez-Mares, 2014; Magalhães et al., 2021). The most common reasons for caffeine consumption include its ability to reduce fatigue during long working hours (Franke et al., 2015), promote wakefulness (Isa et al., 2021; Roehrs & Roth, 2008), and provide energy (Dong et al., 2020). Caffeine has been shown to enhance cognitive performance by improving attention (Rogers & Dernoncourt, 1998) and concentration (Annuar et al., 2023; McLellan et al., 2016). Moreover, caffeine use in the workplace might help individuals feel more satisfied and improve job performance (Khan, 2019; Kun et al., 2023).

Individuals who are addicted to work often experience adverse outcomes, such as sleep and anxiety disorders, partly due to their perfectionistic tendencies (Andreassen et al., 2012; Andreassen, Griffiths, et al., 2016; Falco et al., 2017). These individuals also tend to push themselves to perform better (Shimazu et al., 2015). Such traits may increase the likelihood of work-addicted individuals using stimulants such as caffeine to boost performance and sustain prolonged working hours (Kun et al., 2023). This suggests that, caffeine consumption may be prevalent among individuals addicted to work as a way to cope with intense work demands and stress (Salanova et al., 2016). Therefore, it was hypothesized that work addiction would be positively associated with caffeine addiction (H_4_).

#### Work addiction and nicotine addiction

It has been suggested that nicotine use may have stress and anxiety reducing effects (Benowitz, 2010). Individuals who smoke cigarettes often turn to nicotine to enhance concentration, regulate their performance in work processes, and manage stress levels (Benowitz, 2010; Heishman, 1999; Knott et al., 2011). Research examining work environments indicates that work-related stress can trigger smoking behaviour and nicotine dependence (Son et al., 2016). Individuals who are addicted to work often exhibit tendencies towards high stress and anxiety due to their strong desire for performance excellence (Andreassen et al., 2012; Clark et al., 2014; Griffiths, 2005). These individuals are prone to using stimulants to maintain emotional balance and improve performance during work processes (Kun et al., 2023).

Moreover, both work addiction (Andreassen et al., 2014; Rachubińska et al., 2023) and nicotine addiction (Hakulinen et al., 2015; Kahler et al., 2009; Munafo et al., 2007; Terracciano & Costa, 2004) have been reported to show significant positive associations with neuroticism. In light of these findings, it is anticipated that individuals who are addicted to work may have an increased likelihood of smoking to reduce stress and anxiety and to regulate their work performance. Therefore, it was hypothesized that work addiction would be positively associated with nicotine addiction (H_5_).

## Methods

### Participants and procedure

A total of 693 employees from the public and private sectors, working as blue-collar and white-collar employees, participated in the study. Of these, 48% were male, 44.9% were married, 54.3% had a university degree or higher, 56.6% were white-collar employees, and 30.6% held managerial positions. Regarding their positions in the organizational hierarchy, 24.5% were at the lowest level, 45.9% at the middle level, and 20.3% at the upper level. Among managerial roles, 27.1% were lowest level managers, 27.7% were mid-level managers, and 14.9% were upper-level managers. The participants had a mean age of 31.6 years (SD = 10.60; range = 18–65 years), an average work experience of 10.4 years (SD = 9.93; range = 0–45 years), and an average weekly working time of 39.3 hours (SD = 15.57; range = 8–90 hours).

Data were collected through an online survey distributed through the authors’ online social media networks between December 8 and 29 December 2024. The survey was disseminated using *Google Forms* and targeted employees actively working in various public and private sectors across Türkiye. A total of 790 participants responded to the survey. However, responses from participants who incorrectly answered control questions or provided incomplete and/or careless responses (97 cases) were excluded from the analysis. Therefore, the final sample comprised 693 participants. The survey included questions about work addiction, shopping addiction, social media addiction, caffeine addiction, food addiction, nicotine addiction, and demographic information. Participation was voluntary, and informed consent was obtained from all participants. Ethical approval for the study was obtained from the first author’s university’s ethics board before data collection. The study complied with the principles outlined in the Helsinki Declaration (Ethics approval number: E-61923333-050.99 -428,828).

### Measures

#### Demographics

Participants first answered demographic questions indicating their gender (1 = Male, 2 = Female, 3 = Prefer not to say, 4 = Other), marital status (1 = Married, 2 = Single), education level (1 = Primary school, 2 = Middle school, 3 = high school or equivalent, 4 = Associate degree, 5 = Bachelor’s degree, 6 = Master’s degree, 7 = Doctorate), employment category (1 = Blue-collar, 2 = White-collar), and their positions in the organizational hierarchy (1 = Lowest level, 2 = Middle level, 3 = Upper level). Participants were also asked about their managerial roles (1 = Yes, 2 = No) and managerial positions (1 = Lower-level manager, 2 = Mid-level manager, 3 = Upper-level manager). Open-ended questions were included to assess participants’ age, work experience (in years).

#### Bergen Work Addiction Scale (BWAS)

The BWAS (Andreassen et al., 2012; Turkish version: Özsoy, 2019b) comprises seven items (e.g., *“How often during the last year have you thought of how you could free up more time to work?”)* and assesses work addiction as a single dimension. Participants rate items on a five-point Likert-type scale ranging from 1 (*never*) to 5 (*always*). To determine individuals at high risk for work addiction, a cut-off score was applied, where individuals who scored 4 or 5 on at least 4 out of 7 items were classified as high-risk (Andreassen et al., 2012).

#### Bergen Social Media Addiction Scale (BSMAS)

The BSMAS (Andreassen, Billieux, et al., 2016; Turkish version: Demirci, 2019) comprises six items (e.g., *“How often during the last year have you spent a lot of time thinking about social media or planned use of social media?”*) and assesses social media addiction as a single dimension. Participants rate items on a five-point Likert-type scale ranging from 1 (*very rarely*) to 5 (*very often*). The total score on the scale ranges from 6 to 30. To identify individuals at high risk of addiction, a cut-off score was applied, where individuals scoring 24 or higher were classified as high-risk (Luo et al., 2021).

#### Bergen Shopping Addiction Scale (BSAS)

The BSAS (Andreassen et al., 2015; Turkish version: Aksu, 2023) comprises seven items (e.g., *“I think about shopping/buying things all the time”*) and assesses work addiction as a single dimension. The scale is unidimensional, and participants rate items on a five-point Likert-type scale ranging from 0 (*strongly disagree*) to 4 (*strongly agree*). The total score on the scale ranges from 0 to 28. To identify individuals at high risk of addiction, a cut-off score was applied, where individuals scoring 23 or higher were classified as high-risk (Zarate et al., 2023).

#### Modified Yale Food Addiction Scale version 2.0 (mYFAS 2.0)

The mYFAS 2.0 (Schulte & Gearhardt, 2017; Turkish version: Tok et al., 2023) comprises 13 items (e.g., *“I ate to the point where I felt physically ill”*). Participants rate items on an eight-point Likert-type scale ranging from 0 (*never*) to 7 (*every day*). Higher scores indicate a higher risk of food addiction. The total score on the scale ranges from 0 to 11. To identify individuals at high risk of food addiction, at least two of the 13 items assessing symptoms must be met (one item assessing impairment and the other distress), along with at least six symptoms from the remaining 11 items (Schulte & Gearhardt, 2017). Items scoring 5 or above were considered to indicate that the corresponding symptom was met.

#### Caffeine Use Disorder Questionnaire (CUDQ)

The CUDQ (Ágoston et al., 2018; Turkish version: Kaya et al., 2023) comprises 10 items (e.g., *“Did you feel a strong desire or had unsuccessful attempts to reduce or control your caffeine consumption?”*). Participants rate items on a four-point Likert-type scale ranging from 1 (*never*) to 4 (*very often*). Higher scores indicate a higher risk of caffeine use disorder. The total score on the scale ranges from 10 to 40. Because there is no universally accepted cut-off score for being at risk of caffeine addiction, the determination of the cut-off score was based on the widely used mean + 2 standard deviations approach (Sharma & Jain, 2014). Therefore, individuals who scored 28.5 or above out of 40 were classified as being at high-risk.

#### Fagerström Test for Nicotine Dependence (FTND)

The FTND (Fagerstrom, 2003; Turkish version: Uysal et al., 2004) comprises six items designed to assess nicotine dependence (e.g., *“How soon after waking up do you smoke your first cigarette or use nicotine?”*). Each item has a specific scoring range based on the response options, with total scores ranging from 0 to 10. Participants respond to questions about their smoking habits and consumption patterns, such as time to smoke the first cigarette of the day, daily cigarette consumption, and difficulty refraining from smoking in restricted areas. To identify individuals at high risk of nicotine dependence, a cut-off score of 6 or higher was used, with individuals meeting this threshold being classified as high-risk (Fagerström & Schneider, 1989).

### Data analysis

The data were analysed using the Statistical Package for the Social Sciences (SPSS) version 22 software. Descriptive statistics for participants’ demographic information and difference tests (independent samples *t*-tests, ANOVAs) are presented in Table 1. Descriptive statistics (frequency analyses, means, standard deviations, standard errors) and internal consistency analyses (Cronbach’s alpha, McDonald’s omega, and composite reliability) are summarized in Table 2. Prevalance rates, overlap matrix of high-risk addiction groups, and chi-square results are shown in Table 3. Correlation findings are shown in Table 4.

**Table 1.** Demographic statistics and difference tests findings (*N* = 693).

Variables	Category	N	%	Mean	t (or F)
Gender	Male	330	48	2.84	
	Female	351	51	2.94	2.65
	Did not want to specify	7	1	3.29	
Marital status	Married	309	44.9	2.95	1.65
	Single	379	55.1	2.85	
Education level	Primary school	15	2.2	2.81	
	Middle school	15	2.2	2.47	
	High school and equivalent	202	29.1	2.89	
	Associate degree	78	11.3	2.95	1.40
	Bachelor’s degree	279	40.3	2.91	
	Master’s degree	64	9.2	2.81	
	PhD	33	4.8	3.07	
Employee category	Blue-collar	204	29.4	2.86	−1.67
	White -ollar	392	56.6	2.96	
Organizational position	Lowest level	170	24.5	2.80	
	Mid-level	318	45.9	2.97	3.11*
	Upper level	141	20.3	2.93	
Managerial role	Yes	212	30.6	2.97	
	No	432	62.3	2.89	1.28
Managerial position	Lowest level manager	188	27.1	2.86	
	Middle level manager	192	27.7	3.00	1.82
	Upper level manager	103	14.9	2.90	

*p < 0.05.

Mean = mean score on the bergen Work Addiction Scale.

**Table 2.** Descriptive statistics and internal consistency scores on the psychometric scales.

Variables	Mean	SD	α	ω	CR
Bergen Work Addiction Scale (BWAS)	2.89	0.75	.70	.71	.80
Bergen Social Media Addiction Scale (BSMAS)	2.59	0.93	.81	.81	.87
Modified Yale Food Addiction Scale Version 2.0 (mYFAS 2.0)	1.31	1.31	.92	.92	.93
Bergen Shopping Addiction Scale (BSAS)	1.28	0.82	.85	.85	.89
Caffeine Use Disorder Questionnaire (CUDQ)	1.58	0.64	.91	.92	.93
Fagerström Test for Nicotine Dependence (FTND)	1.41	1.99	.76	.81	.87

*M* = mean, SD = standard deviation, α = Cronbach’s alpha, ω = McDonald’s Omega, CR = Composite reliability.

**Table 3.** Prevalance rates, overlap matrix of high-risk addiction groups and chi-square results.

High-hisk addiction groups based on addiction type	Prevalance (%)	Number of individuals at high-risk	1	2	3	4	5	6
1. Work addiction	28	194	-					
2. Social media addiction	8.8	61	16	-				
3. Food addiction	1.4	10	12	6	-			
4. Shopping addiction	4.3	30	2	2	1	-		
5. Caffeine addiction	5.5	38	15	12***	3	3	-	
6. Nicotine addiction	12.3	85	29	8	7	3	18***	-

Chi-square (χ^2^) test significance: *p < .05, **p < .01, ***p < .001. High-risk categorization does not indicate a formal addiction diagnosis but identifies individuals at potential risk.

**Table 4.** Correlation matrix of study variables.

Variables	1	2	3	4	5	6	7	8	9
1. Work addiction	-								
2. Social media addiction	.06	-							
3. Food addiction	.14***	.36***	-						
4. Shopping addiction	.12**	.43***	.38***	-					
5. Caffeine addiction	.16***	.29***	.37***	.35***	-				
6. Nicotine addiction	.06	.09*	.20***	.10**	.36***	-			
7. Gender	.08*	.11**	.02	.26***	.06	−.16***	-		
8. Marital status	−.06	.23***	.000	.15***	.16***	.03	.06	-	
9. Age	.06	−.28***	−.12**	−.20***	−.22***	−.10*	−.09*	−.62***	-

*p < 0,05, **p < 0,01, ***p < 0,001, Gender (1 = Male, 2 = Female), Marital Status (1 = Married, 2 = Single).

## Results

The demographic distribution of participants is detailed in Table 1. The analysis indicated that work addiction tended to be higher among mid-level employees compared to lower-level employees.

Table 2 presents the study variables’ means, standard deviations, and internal consistency coefficients (Cronbach’s alpha, McDonald’s omega, and composite reliability). The internal consistency coefficients of the variables ranged between good and excellent (0.70–0.92).

Individuals at high-risk of each addiction were identified based on the cut-off scores specified in the respective scales’ descriptions (see “Measures” section). Table 3 presents the prevalence rates of high-risk individuals for each addiction type and the overlap between these high-risk groups. The highest prevalence was observed for work addiction (28%), followed by nicotine addiction (12.3%), social media addiction (8.8%), caffeine addiction (5.5%), and shopping addiction (4.3%), with the lowest prevalence for food addiction (1.4%). Significant overlaps were found in only two instances: (i) individuals who were at high-risk of caffeine addiction were also at high-risk of social media addiction, and (ii) individuals who were at high-risk of nicotine addiction were also at high-risk of caffeine addiction.

Correlation analysis showed statistically significant positive relationships between scores on the work addiction scale and scores on the (i) food addiction scale (*r* = .14), (ii) shopping addiction scale (*r* = .12), and (iii) caffeine addiction scale (*r* = .16). However, no statistically significant relationships were found between work addiction scale and (i) social media addiction scale and (ii) nicotine addiction scale. Based on these findings, H_1_ and H_5_ were rejected, whereas H_2_, H_3_, and H_4_ were supported (Table 4).

## Discussion

The present study examined the relationship between work addiction and five other types of addiction (i.e., social media addiction, food addiction, shopping addiction, caffeine addiction, and nicotine addiction). The findings showed significant relationships between work addiction and three other addictions (i.e., food addiction, shopping addiction, and caffeine addiction). Based on these findings, H_1_ and H_5_ were rejected, and H_2_, H_3_, and H_4_ were supported.

### Interpretation of findings

No statistically significant relationship was found between work addiction and social media addiction (on either the correlation between scale scores (Table 4) or among the groups of overlapping high-risk individuals (Table 3). Therefore, H_1_ was not supported. Social media use serves a variety of purposes, including entertainment and leisure, following trends and updates, emotional relaxation, satisfying the need for approval, product research and shopping, self-promotion, image management, and establishing connections (Andreassen & Pallesen, 2014; Przybylski et al., 2013). However, individuals addicted to work may have more limited and distinct motivational reasons for using social media. These individuals are more likely to use social media for professional purposes, such as staying updated on work-related developments, promoting their achievements, and increasing their visibility (Falco et al., 2017). Moreover, work-addicted individuals may address these needs by utilizing other online tools, such as email, work forums, or professional networking platforms, rather than engaging in social media use (Horváth et al., 2024). These factors may explain the absence of a significant relationship between work addiction and social media addiction.

A significant positive relationship was found between work addiction and shopping addiction (based on scale scores (Table 4), supporting H_2_, but not among the groups of overlapping high-risk individuals (Table 3). Individuals addicted to work may experience a stressful lifestyle due to an intense work pace and constant occupational engagement. For these individuals, shopping may serve as a short-term relief (Mücevher & Gül, 2023), fulfilling needs for instant gratification and emotional satisfaction (Arnold & Reynolds, 2003; Weinstein & Lejoyeux, 2010). Additionally, individuals may reward themselves by quickly shopping through digital platforms while at work or during work-related activities (Cai & Cude, 2016). These factors make shopping more accessible and appealing for individuals who are addicted to work. Additionally, individuals with a high tendency towards shopping addiction may allocate more time and energy to work to compensate for potential financial losses or repay their debts. This possibility suggests that the relationship between work addiction and shopping addiction may be reciprocal to some extent.

A significant positive relationship was found between work addiction and food addiction (based on scale scores (Table 4), supporting H_3_, but not among the groups of overlapping high-risk individuals (Table 3). Individuals addicted to work may engage in eating behaviours to fulfil their needs for emotional relief and instant gratification during long working hours. Due to the extensive time devoted to work and the motivation to save time, these individuals may tend to consume convenience foods and snacks more frequently, which can result in food addiction. Additionally, individuals with high levels of work addiction may neglect sleep or experience sleep disturbances due to excessive time devoted to work (Salanova et al., 2016). This can lead to an increase in ghrelin levels, a hormone associated with hunger, resulting in a preference for high-calorie foods (Spiegel et al., 2004). Furthermore, individuals under high stress and time pressure may develop unhealthy eating habits to achieve quick satisfaction (Hauck et al., 2020). Moreover, the stress associated with work addiction may elevate cortisol levels, which in turn can heighten cravings for carbohydrate- and fat-rich foods, potentially triggering food addiction (Adam & Epel, 2007). These physiological and behavioural mechanisms, when combined with dysregulation in reward and motivation systems, can reinforce addictive tendencies (Fekih-Romdhane et al., 2022).

A significant positive relationship was found between work addiction and caffeine addiction based on scale scores (Table 4), supporting H_4_, but not among the groups of overlapping high-risk individuals (Table 3). Fatigue, sleep disturbances, anxiety disorders, and stress, which often result from long working hours and the effort to achieve better performance, are among the most well-known consequences of work addiction (Andreassen et al., 2012; Andreassen, Griffiths, et al., 2016; Griffiths, 2005). Individuals addicted to work may consume more caffeine both to cope with these issues and to benefit from the effects of caffeine, such as reducing fatigue, promoting wakefulness, and providing energy (Dong et al., 2020; Franke et al., 2015; Isa et al., 2021; Roehrs & Roth, 2008).

No statistically significant relationship was found between work addiction and nicotine addiction (on either the correlation between scale scores (Table 4) or among the groups of overlapping high-risk individuals (Table 3). Therefore, H_5_ was not supported. This may be attributed to several factors. One explanation could be the restrictive regulations regarding smoking policies within workplaces. Another explanation is that work addiction, which is often associated with psychosocial factors such as achievement orientation, perfectionism, and the need for social approval (Andreassen et al., 2012; Clark et al., 2014; Griffiths, 2005; Kun et al., 2023), differs from nicotine addiction, which, despite its psychological implications, is also rooted in biological and chemical mechanisms (Benowitz, 2010; Heishman, 1999; Kutlu et al., 2015). Smoking in work environments could lead to negative reactions from supervisors or colleagues, potentially discouraging individuals from smoking. Additionally, individuals with work addiction may perceive smoking as a waste of time due to their strong work focus, be inclined to avoid creating a negative impression on their family and social circles, and exhibit a lower likelihood of nicotine use because they tend to spend their non-working hours engaging in work-related activities. Therefore, the two types of addiction may stem from different motivations and processes.

In the present study, the hypotheses were tested based on the correlations between the scores on the addiction scales. However, it should be noted that the correlations between scores on the scales are not necessarily correlations between addictions. Individuals can score high (but below addiction cut-offs) on the scales. Moreover, the results indicated that when an individual was classified as being at high-risk for a behavioural addiction, the likelihood of concurrently having a substance- or food-based addiction was significantly higher than that of another behavioural addiction. Similarly, the findings suggest that two substance-based addictions (e.g., caffeine and nicotine) may co-occur.

The results also indicated a significant association between individuals who were at high-risk for both (i) caffeine addiction and social media addiction, and (ii) caffeine addiction and nicotine addiction (i.e., individuals who scored above the cut-offs for being at high-risk of addiction on more than one potentially addictive behaviour). These findings suggest that while the probability of being genuinely addicted to two behavioural addictions simultaneously is low, the likelihood of a substance-based addiction (e.g., caffeine) co-occurring with a behavioural addiction is much higher. This association is plausible, because individuals who spend excessive amounts of time on social media can consume high amounts of caffeine simultaneously to maintain focus or combat fatigue. For instance, an individual might engage in social media activity while consuming caffeinated beverages, demonstrating the compatibility of these behaviours. Moreover, the co-occurrence of caffeine and nicotine addiction is also highly probable, given their frequent joint consumption in daily life. The simultaneous engagement in these behaviours highlights their potential for comorbidity.

### Limitations and future research suggestions

The present study has several limitations. First, the data were collected using self-report measures. Consequently, specific characteristics of the self-report method (e.g., high social desirability, low self-awareness, memory distortions) may introduce biases in the results. Second, the average age of participants in the study was 31.4 years, suggesting that the sample was predominantly young adults, and therefore not representative of the Turkish population. Third, a non-probability sampling method, specifically convenience sampling, was employed to ensure an adequate number of participants during the data collection process, and therefore (again) was not representative of the Turkish population. Fourth, the study’s cross-sectional design limited the ability to determine causality between the study variables or examine how potentially addictive behaviours may change over time. Finally, the study was conducted in Türkiye, which limits the generalizability of the findings to different cultural contexts. Based on these limitations, future longitudinal studies should be conducted with larger and more representative samples from both in and outside of Türkiye to draw more generalizable conclusions.

Despite these limitations, the present study contributes to and extends the existing knowledge regarding the relationship between work addiction and other types of addiction. Future research could examine additional factors that may influence the observed relationships to gain a more comprehensive understanding of the relationships between work addiction and other addiction types. These factors might relate to individual characteristics (e.g., personality traits, coping styles and mental health conditions), organizational settings (e.g., job demands, organizational culture, and leadership styles), or broader social contexts (e.g., cultural differences, economic factors, and technological or digital influences). Lastly, replicating the present study with samples from different cultural backgrounds is recommended to validate and expand the findings.

## Conclusion

The present study was conducted to examine the relationship between work addiction and other types of addiction. The findings showed significant positive relationships between work addiction and three other addictions (i.e., food addiction, shopping addiction, and caffeine addiction). However, these findings were derived from correlations between the scale scores for each of the six potentially addictive behaviours. Comorbidity among individuals classified as high-risk for more than one addiction was only observed between two substance-based addictions (caffeine and nicotine), and between one substance-based addiction (caffeine) and one behavioural addiction (social media). This suggests a low likelihood of two behavioural addictions being genuinely present simultaneously in the same individual. On the other hand, the findings indicate that the simultaneous occurrence of a substance-based addiction with a behavioural addiction, or two substance-based addictions, is more plausible. These results underscore the complex interplay between addiction types and highlight the importance of distinguishing between behavioural and substance-based addictions when interpreting comorbidity.

Given the limited number of studies in the literature that have investigated the relationship between work addiction and other types of addiction and the absence of prior research on this topic within the Turkish context, the present study makes a notable contribution to the literature. Moreover, the findings may inform future studies on the antecedents of addiction types, their interrelations, and their connections with individual, behavioural, and environmental factors. These findings can also guide future research exploring these relationships across different cultural contexts to enhance understanding of the shared mechanisms underlying addictions.

## Data Availability

The dataset generated during and analysed during the present study are available from the corresponding author upon reasonable request.
